# Mate-Searching Behaviour of Common and Rare Wasps and the Implications for Pollen Movement of the Sexually Deceptive Orchids They Pollinate

**DOI:** 10.1371/journal.pone.0059111

**Published:** 2013-03-11

**Authors:** Myles H. M. Menz, Ryan D. Phillips, Kingsley W. Dixon, Rod Peakall, Raphael K. Didham

**Affiliations:** 1 Kings Park and Botanic Garden, The Botanic Gardens and Parks Authority, Perth, Western Australia, Australia; 2 School of Plant Biology, The University of Western Australia, Perth, Western Australia, Australia; 3 Evolution, Ecology and Genetics, Research School of Biology, The Australian National University, Canberra, Australian Capital Territory, Australia; 4 School of Animal Biology, The University of Western Australia, Perth, Western Australia, Australia; 5 Commonwealth Scientific and Industrial Research Organisation (CSIRO) Ecosystem Sciences, Centre for Environment and Life Sciences, Perth, Western Australia, Australia; University of Sydney, Australia

## Abstract

Pollinator behaviour directly affects patterns of pollen movement and outcrossing rates in plants. In orchids pollinated by sexual deception of insects, patterns of pollen movement are primarily determined by the mate-searching behaviour of the deceived males. Here, using a capture-mark-recapture study (CMR) and dietary analysis, we compare mate-searching behaviour in relation to local abundance of two pollinator species and explore the implications for pollen movement in sexually deceptive *Drakaea* (Orchidaceae). *Drakaea* are pollinated solely by the sexual deception of male thynnine wasps. The rare *Drakaea elastica* and widespread *D. livida* occur sympatrically and are pollinated by the rare but locally common *Zaspilothynnus gilesi*, and the widespread and abundant *Z. nigripes*, respectively. Local abundance was significantly different with *Z. nigripes* twice as abundant as *Z. gilesi*. For the 653 marked wasps, there was no significant difference in median movement distance between *Z. gilesi* and *Z. nigripes*. However, the maximum movement distance was twice as high for *Z. gilesi* (556 m) compared with *Z. nigripes* (267 m). This is up to three times greater than previously reported for thynnines in CMR studies. Recapture rates were six times higher in *Z. gilesi* (57%) compared to *Z. nigripes* (9%). Pollen loads and wasp longevity were similar, suggesting that this difference in recapture rate arises due to differences in the number of males moving at a scale >500 m rather than through diet or mortality. Differences in the frequency of longer movements may arise due to variation in the spatial distribution of the wingless females. We predict that pollen movement will largely be restricted to within populations of *Drakaea* (<500 m), with few movements between populations (>500 m).

## Introduction

Pollinator behaviour directly influences patterns of pollen movement and the rate of outcrossing in plants, which are important for gene flow and seed fitness [1], [2]. Pollination by food-foraging animals predominantly leads to short distance pollen movement, as they tend to move between neighbouring flowers or inflorescences [3], [4]. On the other hand, pollen movement patterns may be different for pollinators displaying territorial and courtship behaviour compared with food foraging behaviour. Consequently, plants pollinated by sexual deception offer an interesting opportunity to test the consequences of mate-searching behaviour on pollen movement [5].

Pollination by sexual deception is one of the most specialised pollination systems. It involves the attraction of male insects by floral mimicry of the species-specific sex pheromones of female insects [6], [7]. Pollination is achieved when male insects are sexually attracted to the flower, often involving attempted copulation with the labellum, which brings them into contact with the pollinium and stigma [8], [9]. Sexual deception is primarily restricted to the Orchidaceae, with the exception of recently discovered cases from a South African daisy [10] and a European iris [11]. Sexual deception has been confirmed in orchids from Australia, Europe, Central and South America and southern Africa, utilising a wide taxonomic diversity of insect pollinators, within the Hymenoptera and Diptera [12]. Australia is recognised as one centre of diversity of sexually-deceptive pollination systems with at least 150 confirmed cases involving multiple orchid genera [12]–[15], the vast majority of which are pollinated by thynnine wasps [12], [15]. Thynnine wasps represent a diverse, predominantly Australian insect group, with an estimated 1500-2000 species, many of which are undescribed [13]. Male thynnines patrol in search of wingless females that they carry *in copula* to a food source, usually nectar or exudations from scale insects (Hemiptera: Psyllidae) [16]–[18].

In species pollinated by sexual deception, patterns of flower visitation and pollen movement are predicted to reflect the optimal mate-searching behaviour of the pollinating male insect [5]. Intense male competition arises from the low number of sexually receptive females relative to males at any point in time [19]–[21] and is a characteristic of thynnine wasps. Consequently, male insects may search in such a way as to increase their chance of encountering unmated females [21], [22]. However, the mate-searching behaviour adopted may be partially determined by the spatial distribution of females. For example, if females are patchy in the landscape, then males may devote more time to searching in areas where females have previously emerged. By contrast, when females are more uniformly-distributed males may search more evenly in the landscape. To test if mate-searching behaviour of thynnines is affected by local abundance requires studying sympatric species of similar size with similar ecological requirements.

Here we compare pollinator mate-searching behaviour in relation to local pollinator abundance and explore the potential implications for pollen movement in sexually deceptive *Drakaea* (Orchidaceae). We supplement this with a dietary study of the pollinators to ensure that movement patterns and local abundance are not simply affected by differences in diet. *Drakaea elastica* Lindl. and *Drakaea livida* J.Drumm. occur sympatrically in southwestern Australia, but *D. elastica* is rare and threatened while *D. livida* is relatively common [23]. *Drakaea elastica* is pollinated by the rare but locally common *Zaspilothynnus gilesi* Turner (Hymenoptera: Thynnidae), while *D. livida* is pollinated by the widespread and abundant *Z. nigripes* Guerin [24]. The wasps themselves have no dependence or feeding association on *Drakaea* orchids, and are only attracted to orchids by sexual deception. We predict that the rare *Z. gilesi* will display more restricted movement patterns, whereas the common *Z. nigripes* will show a more even distribution of movement distances.

## Methods

### Study system

**Figure 1:**
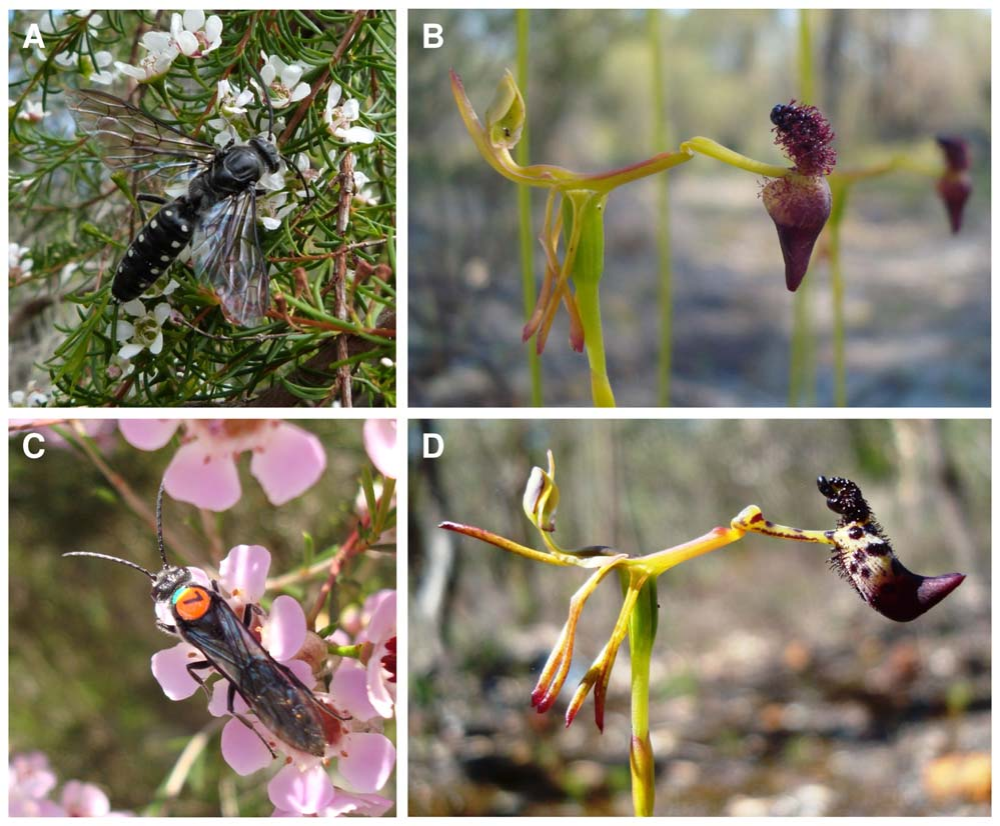
Male *Zaspilothynnus* wasps and the *Drakaea* they pollinate. (A) The thynnine wasp *Zaspilothynnus gilesi*, the pollinator of the rare *Drakaea elastica* (Orchidaceae) (B), feeding on nectar of *Astartea fascicularis* (Myrtaceae); (C) *Zaspilothynnus nigripes*, the pollinator of the common *Drakaea livida* (Orchidaceae) (D), marked with green paint pen and an orange queen bee tag, feeding on *Chamelaucium uncinatum* (Myrtaceae). Photographs: (A), (B) and (D) MHM Menz, (C) J Hardwick.

We used a capture-mark-recapture (CMR) study to compare the mate-searching behaviour of the rare *Z. gilesi* and the common *Z. nigripes* (Figure 1). Both species of *Zaspilothynnus* are within the same size range (∼20-30 mm in length). They frequently co-occur and have a similar flight phenology, allowing direct comparison of mate searching behaviour. The few thynnines for which reproduction has been studied have been found to be parasitoids on scarab beetle larvae (Coleoptera: Scarabaeidae) [16]-[18], [25]–[27]. The apterous females spend most of their lives underground, only emerging to attract a male [17], [26], [27]. Consequently, there is always an excess of males ready to mate relative to females. Nectar-feeding thynnines are most often observed feeding on open-flowered plants such as many Myrtaceae that are accessible to a broad array of pollinators [15], [28].

Within continuous habitats, *Drakaea* typically form scattered sub-populations (<500 m), restricted to low-lying open sandy microhabitats, leading to a naturally patchy distribution [29]. The rare *Drakaea elastica* is endemic to the Swan Coastal Plain, Western Australia, and is listed as critically endangered under the Australian Federal Environmental Protection and Biodiversity Conservation Act (EPBC) [30], whereas *D. livida* is widespread and relatively common in the south-west of Western Australia [23] (Figure 1). *Drakaea elastica* is restricted to areas of grey sand within mixed *Banksia* spp. and *Kunzea glabrescens* Toelken woodland [30]. Natural vegetation in this region is highly fragmented due to land clearing for housing development and agriculture [31]. As a result, the majority of the remaining populations of this species are small (<50 plants) [32] and disjunct (>1 km apart). In many of these populations, *D. elastica* occurs sympatrically with *D. livida*.

### Study site

The CMR study was carried out between 7 October and 3 November 2010 in a remnant *Banksia* spp. and *Kunzea glabrescens* woodland approximately 12 km south-west of Capel (33°38′36″S, 115°29′54″E), Western Australia. Willow peppermint trees, *Agonis flexuosa* (Willd.) Sweet, contributed to a large part of the overstorey, with cover values of up to 70% in the northern half of the study area. The 80×450 m study site was a portion of a longer, linear habitat remnant connected to a reserve otherwise surrounded by pastures. The study site was selected because (i) the rare *Z. gilesi* and common *Z. nigripes* were found to co-occur there in relatively large numbers, based on preliminary site-selection surveys, and (ii) there were no naturally-occurring *Drakaea* orchids at the site (thus avoiding potential interference from orchid-wasp interactions at orchids growing *in situ*). The remnant has an open understorey and prior to the CMR trial males of both wasp species were observed to patrol regularly in search of females throughout the study site.

### Ethics statement

All necessary permits to conduct this research were obtained from the Western Australian Department of Environment and Conservation. No animal ethics approval was required for this research.

### Capture, marking and recapture

Wasps were attracted using the baiting method developed by Stoutamire [33] and Peakall [8], where fresh orchid flowers are presented for short time intervals to lure male wasps. Orchids were picked and stored in a portable refrigerator. Depending on fresh flower availability, baiting for the two thynnine species *Z. gilesi* and *Z. nigripes* was undertaken simultaneously, with orchid baits placed at 108 and 88 random locations respectively, over the 27 day study period. Each location was only baited once for 10 minutes. When both orchid species were used simultaneously, they were placed approximately 30 cm apart. Baiting was restricted to warm (>18°C) and sunny days between 09:00 and 16:00 hrs, when thynnine wasps are most active [8]. Up to 10 locations were visited per day, with a minimum distance of 30 m between consecutive locations visited. Bait flowers were kept in a sealed container between baiting locations to avoid wasps following the flowers and to overcome habituation to the floral odour by the wasp.

When arrival rates of wasps at the orchid baits were relatively low (1-3 individuals in a 10-minute period), individuals were marked on the thorax with a queen bee tag (2.5 mm diameter), attached using the glue provided with the tags (sourced from: http://www.penders.net.au). When arrival rates at the orchid baits were higher (4-32 individuals in a 10-minute period) it was not feasible to tag individuals on first capture using queen bee tags. Instead, each individual was marked on the thorax using one or a combination of coloured paint pen (Uniball Posca) marks unique to each location. Upon first recapture, the paint pen mark was supplemented with a queen bee tag. There was no loss of information in this process as all single- and multi-coloured paint pen marks were clear at recapture allowing accurate identification of origin. After marking, wasps were released at the point of capture and observed to fly away from the area until lost from sight (>10 m). Marking of individuals continued for the duration of the study.

Sweep-netting for patrolling males was conducted for twelve 15-minute periods on a single day following the baiting. Sweep-netting sites were approximately 30 m apart along a sandy track running NW-SE through the study area, and extended into adjacent woodland for up to 250 m to the NW and SE beyond the boundary of the baiting area.

No baiting was conducted in adjacent pasture during the study, as the two species of thynnine wasp were not observed to mate-search in these areas. However, to confirm this for *Z. gilesi* at the study site, we conducted 48 baiting trials with *D. elastica* in the adjacent pasture up to 400 m from the study remnant in November 2011. This resulted in just a single male *Z. gilesi* being detected.

### Movement distances and patrolling range

Locations of marking and recapture points were recorded using a hand-held GPS. A *G*-test was used to test for a difference in the distribution of recapture frequencies between *Z. gilesi* and *Z. nigripes* (individuals marked but not recaptured, or recaptured 1, 2, or 3-7 times). Mann-Whitney *U*-tests were used to test for a difference in median abundance of wasps per minute across baiting locations, and median number of days between marking and final recapture. All analyses were conducted in GenAlEx 6.5 [34], [35] unless otherwise specified.

Distances between mark and recapture site were calculated in ArcView v3.3 (ESRI Inc.). To test for differences in the frequency distributions of movement distances between *Z. gilesi* and *Z. nigripes*, we implemented a kernel density estimate comparison procedure using the kde.compare function [36] in R v2.14.0 [37] which was adapted from the sm.density.compare function from the R package ‘sm’ [38]. Kernel density estimation is a non-parametric procedure that produces a smoothed estimate of the frequency distribution of movement distances for each species. The kde.compare function compares the area between these two curves to the areas between pairs of curves resulting from a user-specified number of random permutations (here 5000) of the species labels in the data using the sm.density.compare function [38]. The kde.compare function expands on the sm.density.compare function by incorporating automatic bandwidth estimation for the kernel density estimates via the Sheather-Jones bandwidth estimation procedure [39], as implemented in the R function ‘dpik’ from the package ‘KernSmooth’ [40], and increasing the number of points evaluated to produce the accompanying plot thereby resulting in a more detailed figure. Significance is calculated as the percentage of the random permutations that yield an area between the curves greater than that between the curves estimated from the data as grouped by species. Mann-Whitney *U*-tests were used to compare the median distances moved between *Z. gilesi* and *Z. nigripes* for baiting-only recapture records, and baiting combined with sweep-netting records.

### Pollinator diet

Male wasps were swabbed for pollen to determine (i) which food plants the wasps were visiting and (ii) if both species were visiting the same food plant species. During the capture-mark-recapture experiment, 20 males of each species were swabbed for pollen using a gel containing fuchsin stain, as described by Wooller *et al*. [41]. Each wasp was swabbed on the top and underside of the thorax. Pollen carried by the wasps was identified using a compound microscope by comparing samples to a reference collection of pollen from plants flowering at the study site. Pollen load on each wasp was categorised by estimating the number of grains from each plant species, as 1, 1-10, 10-100, 100-1000, and ≥1000 grains. Only plant species represented by ≥10 pollen grains on an individual wasp were considered in the dietary analysis. Relative loads of each pollen species were compared between wasp species using *G*-tests.

## Results

### Marking and recapture

**Figure 2:**
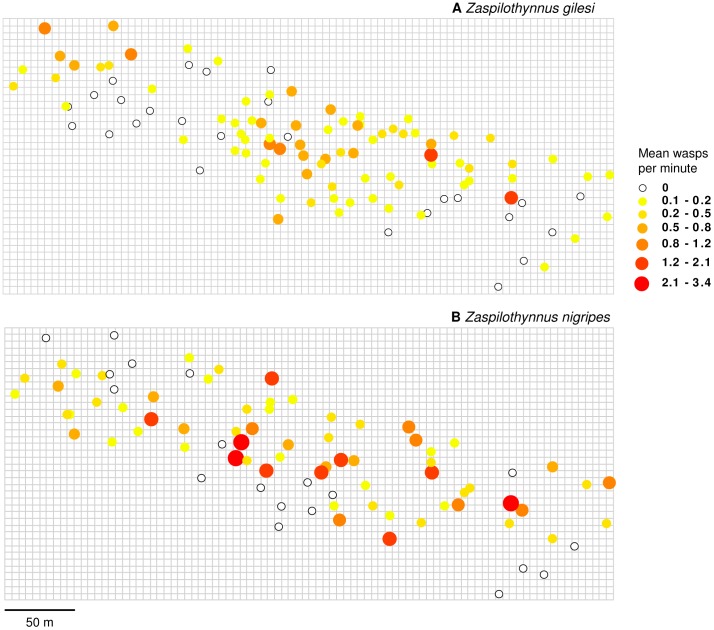
Mean number of male *Zaspilothynnus* wasps per minute attracted to orchid bait flowers. (A) *Zaspilothynnus gilesi* and (B) *Zaspilothynnus nigripes*. Open circles represent baiting locations where no wasps were caught.

We captured and marked a total of 147 individuals of the rare *Z. gilesi* from 108 baiting trials (22% zero responses; range: 0-11 wasps per trial), and 506 individuals of the common *Z. nigripes* from 88 baiting trials (17% zero responses; range: 0-32 wasps per trial), spread over 14 individual days of sampling. In the 88 paired experiments there were no cases where a wasp alighted on an orchid other than the species it typically pollinates (*N* responses  =  653). Considering only locations where wasps were detected, median abundance of *Z. gilesi* (0.2 wasps per minute) was significantly lower than *Z. nigripes* (0.4 wasps per minute) (*Z*  =  3.385, *P*  =  0.001; Figure 2).

For *Z. gilesi*, we recaptured 83 of the 147 marked individuals (56%), with 46 (55%) of these recaptured once, 23 (28%) recaptured twice, eight (10%) recaptured three times, and six (7%) recaptured four to seven times (144 recapture events in total, from the baiting trials and sweep-netting combined). The median time between capture and final recapture for *Z. gilesi* was six days (range 0-23 days).

For *Z. nigripes*, we recaptured 48 of the 506 individuals (9%), with 36 (75%) of these recaptured once, eight (17%) recaptured twice, one (2%) recaptured three times, and three (6%) recaptured four to five times (68 recapture events in total, from the baiting trials and sweep-netting combined). The median time between capture and final recapture for *Z. nigripes* was nine days (range 0-25 days).

**Figure 3:**
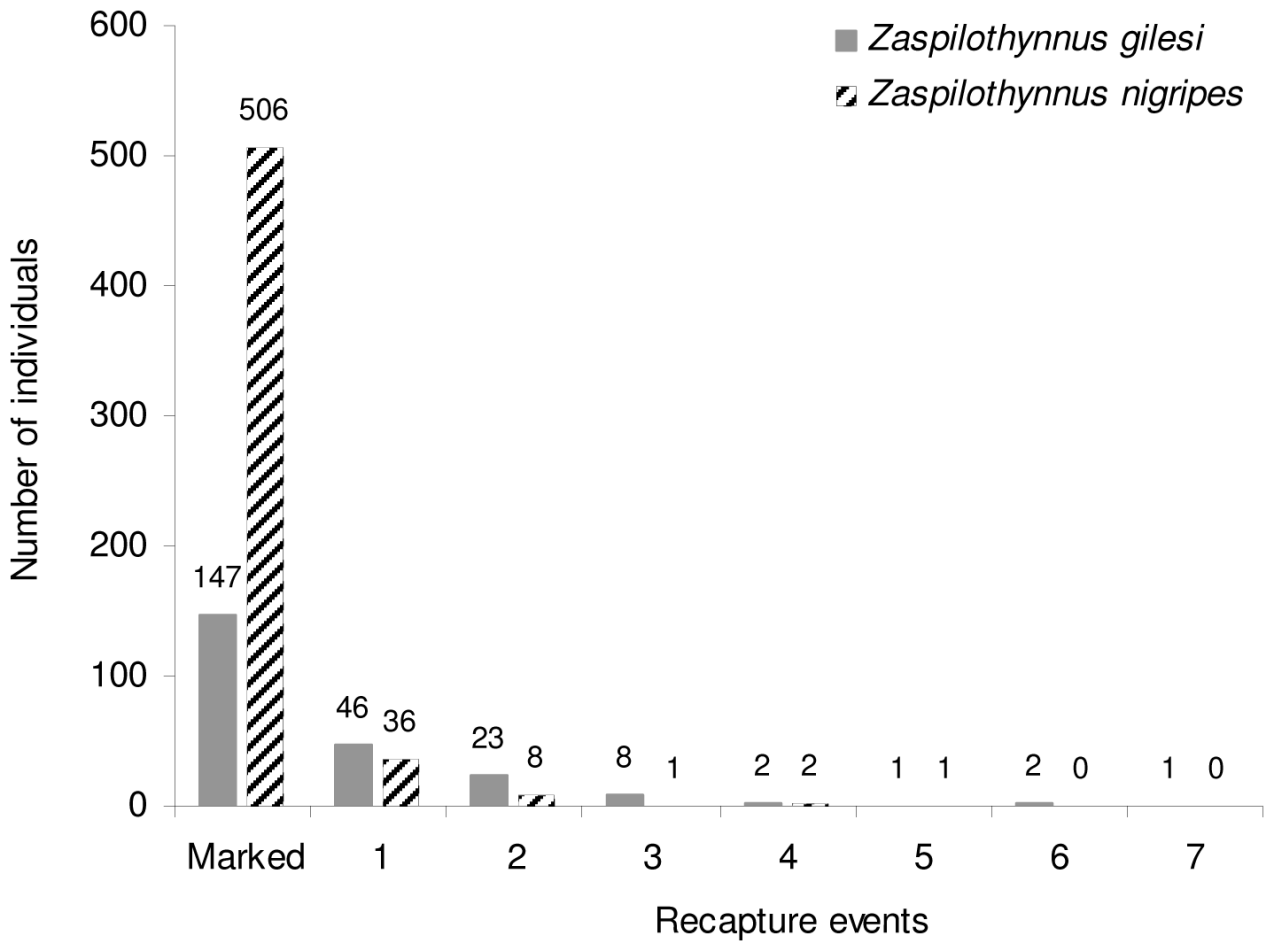
Comparison of the number of male *Zaspilothynnus* wasps marked and the number of times recaptured.

The distribution of recapture frequencies (*G*-test; individuals marked but not recaptured, or recaptured one, two, or three to seven times) differed significantly between the rare *Z. gilesi* and the common *Z. nigripes* (*G*  =  141.12, d.f.  =  3, *P*  =  0.001; Figure 3), with *Z. gilesi* recaptured more frequently. Interestingly, the proportion of same day recaptures was markedly higher for *Z. gilesi* (45%) compared to *Z. nigripes* (5%). Median number of days between marking and final recapture was significantly higher for *Z. nigripes* than *Z gilesi* (*Z*  =  3.46, *P*  =  0.001). This indicates that the lower recapture rate in *Z. nigripes* is unlikely to be due to higher mortality.

### Movement distances

**Figure 4:**
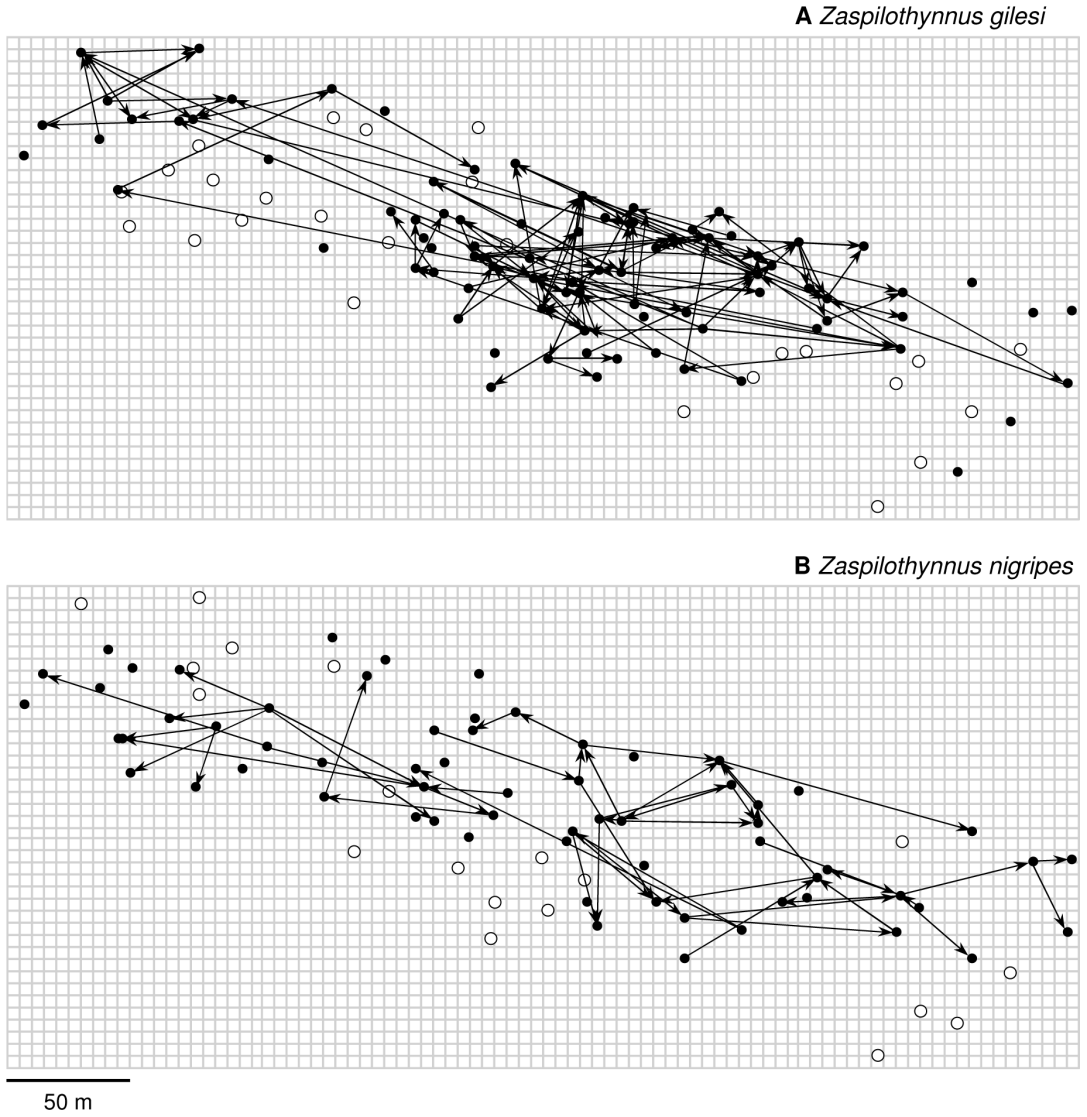
Movement vectors of male *Zaspilothynnus* wasps attracted to orchid bait flowers. (A) *Zaspilothynnus gilesi* and (B) *Zaspilothynnus nigripes*. Open circles represent baiting locations where no wasps were caught.

**Figure 5:**
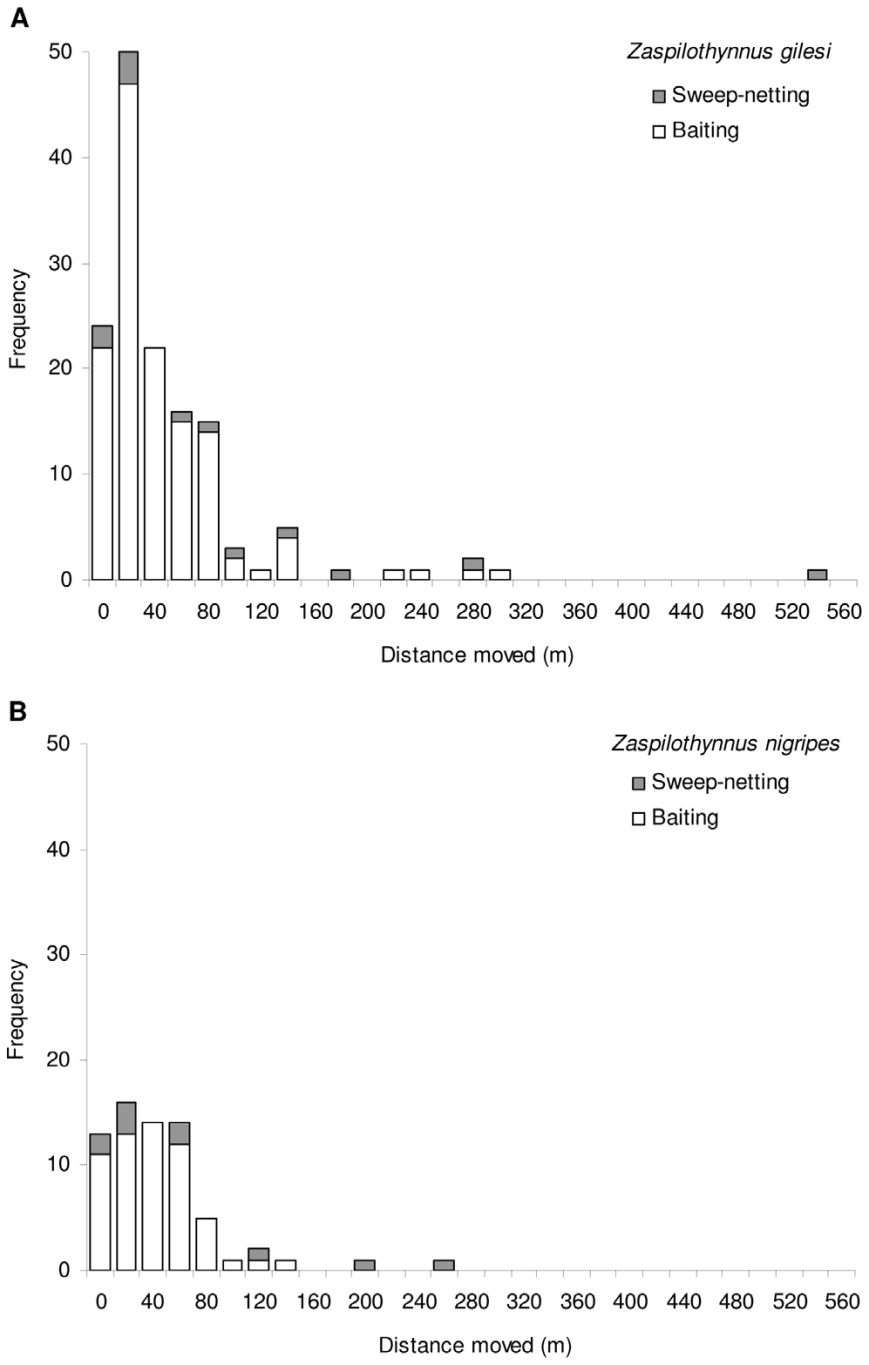
Movement distances of male *Zaspilothynnus* wasps attracted to orchid bait flowers. (A) *Zaspilothynnus gilesi* and (B) *Zaspilothynnus nigripes*. Open bars represent movements recorded from baiting trials and shaded bars from sweep-netting.

**Figure 6:**
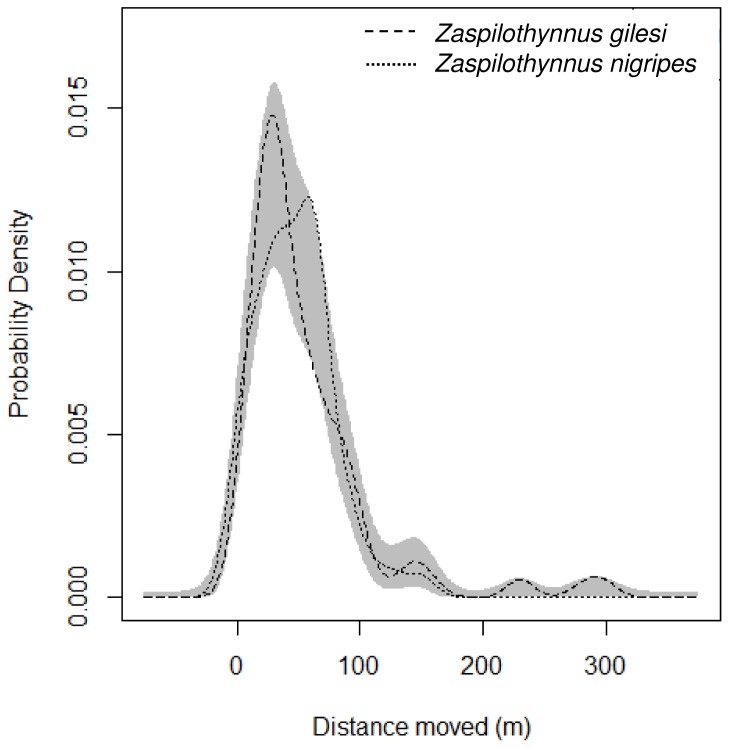
Probability density plots of movement distances of male *Zaspilothynnus* wasps lured to orchid baits. The shaded area represents a null model of no difference between the two frequency distribution curves. Significance values are based on 5000 random permutations.

For *Z. gilesi*, recapture distances for the baiting trials ranged from 0-300 m (median  =  38 m, *N*  =  132), and 0-556 m (median  =  81.5 m, *N*  =  12) from the sweep-netting (Figures 4a and 5a). For *Z. nigripes*, recapture distances ranged from 0-150 m (median  =  46.5 m, *N*  =  58) for the baiting trials, and 0-267 m (median  =  49.5 m, *N  = * 10) from the sweep-netting (Figures 4b and 5b). The median recapture distances from both the baiting trials and the sweep-netting did not differ significantly between the rare *Z. gilesi* and the common *Z. nigripes* (*Z*  =  0.01, *P*  =  0.99; *Z*  =  0.59, *P*  =  0.55 respectively). Based on the kernel density comparison procedure [36], frequency distributions of recapture distances of *Z. gilesi* and *Z. nigripes* were not significantly different (Figure 6; baiting only, *P*  =  0.098; baiting and sweep-netting, *P*  =  0.127).

### Pollinator diet

Only three plant species were represented by ≥10 pollen grains on any one individual. These were *Agonis flexuosa*, *Eucaluptus marginata* Sm. and an unidentified herbaceous species of Asteraceae. Of the 40 wasps sampled, 38 carried mixed pollen loads with ≥10 pollen grains from multiple plant species. Of the 20 *Z. gilesi* sampled, 90% were carrying *E. marginata* pollen, 100% were carrying *A. flexuosa* pollen, and 5% were carrying Asteraceae pollen. Of the 20 *Z. nigripes* sampled, 100% were carrying *E. marginata* pollen, 100% were carrying *A. flexuosa* pollen, and 5% were carrying Asteraceae pollen. Pollen load frequencies for both *E. marginata* and *A. flexuosa* were not significantly different between the two wasp species (*G*  =  0.633, d.f.  =  1, *P*  =  0.89; *G*  =  0.038, d.f.  =  1, *P*  =  0.99, respectively).

## Discussion

Using a capture-mark-recapture (CMR) framework, we tested whether two sympatric orchid pollinators exhibit different patterns of local abundance, mate-searching behaviour and nectar resource use. For both species the majority of movements were concentrated in localized areas, indicating high fidelity to patrolling areas during mate searching. This confirms previous observations of male thynnines repeatedly patrolling set paths [42]. The median movement distances of 38 m and 46.5 m (*Z. nigripes* and *Z. gilesi*, respectively; length  =  20 - 30 mm) are greater than that previously documented for smaller bodied (<20 mm) thynnine pollinators of sexually deceptive orchids (14.8-24 m) [5], [8], [43]. Further, the maximum distance moved (556 m, *Z. gilesi*) was approximately three times greater than previously reported for thynnines in a CMR study (161 m, *Neozeleboria cryptoides*, length  =  9 mm) [43]. In contrast, the bee *Colletes cunicularius* (length  =  10 mm), the pollinator of four species of European *Ophrys* orchids [44], moved on average only 5.2 m in search of mates in the vicinity of a large nesting aggregation [45]. These comparisons demonstrate that mate-searching behaviour of pollinators and subsequent pollen movement will vary between sexually deceptive systems [45].

Despite similar movement distributions, there were also significant differences in recapture rate and the number of same-day recaptures between the two species. The abundant *Z. nigripes* had a significantly lower recapture rate (9%) compared to the less abundant *Z. gilesi* (57%). Given that there was no evidence for differences in mortality, this lower recapture rate may be due to male *Z. nigripes* searching for females at a scale of >500 m (representing the extent of the study area). This may be due to variation in distributions of females and resources in the landscape between the two species. Male insects are predicted to display an optimal mate-search strategy that increases their chance of encountering unmated females [5], [21], [22]. If females are rare or patchy in the landscape, then males may devote more time to searching in areas where females have previously emerged, with occasional longer distance movements in search of females elsewhere. Conversely, in the case of common species such as *Z. nigripes*, females may be more uniformly encountered in the landscape, meaning that mate-searching/patrolling of males is less restricted to a particular local area.

The observation of markedly higher same-day recaptures for *Z. gilesi* compared to *Z. nigripes*, despite similar movement distributions, could be due to male *Z. nigripes* learning to avoid the orchid mimic. The thynnine wasp *N. cryptoides* is known to exhibit short-term (≤24 hours) patch avoidance following attraction to a synthetic pheromone bait [46]. In periods of low female availability, such as may characterise *Z. gilesi*, males may more readily respond to the orchid, regardless of the accuracy of the pheromone or morphological mimicry. This could be experimentally tested by presenting captive male wasps with orchid flowers, in the presence or absence of females. Additionally, response to male competition could be tested by presenting orchid flowers to males at varying orchid to male ratios. Direct comparison of multiple thynnine species under a controlled environment may elucidate interspecific differences in male learning and response to orchid flowers.

Patterns of local abundance were significantly different between *Z. gilesi* and *Z. nigripes*. Utilisation of different food sources could potentially lead to variation in the local abundance of pollinators. However, dietary analysis in this study showed that both wasps were visiting the same plant species as nectar sources. This suggests that different nectar preferences are unlikely to be responsible for the observed differences in local abundance between *Z. gilesi* and *Z. nigripes*. The visitation of *A. flexuosa* and *E. marginata* supports the observation that larger-bodied thynnines regularly feed on nectar from open-flowered Myrtaceae [15]. While frequent visitation of *Agonis flexuosa* may be partially due to its high abundance at the study site, *E. marginata* was rare at the study site. Given the similarity in plant species visited, differences in larval host availability may be more important in regulating local abundance of these parasitic wasps than nectar availability.

### Consequences for pollinator ecology and pollen movement in rare orchids

We found no difference in nectar plant visitation between the two species, suggesting that another part of the lifecycle is limiting the abundance of *Z. gilesi*. There have been very few studies on host choice in thynnines [18], [26], [27], and no studies of the host associations of *Zaspilothynnus* wasps. Given the similarity in other aspects of their ecology, availability of suitable larval hosts could be contributing to the difference in overall abundance between *Z. gilesi* and *Z. nigripes*. For example, *Z. nigripes* may be less host-specific than *Z. gilesi*, or might utilise a far more common and widespread host. Thus, host specificity and abundance may play a major role in limiting populations of *Z. gilesi* and other rare thynnine species and requires further investigation.

Investigation of the behaviour and ecology of thynnine wasps yields insights that are relevant for the conservation of *Drakaea* orchids. Our study of the movement patterns of *Z. gilesi* revealed that the majority of *D. elastica* pollen movement will be within 100 m, with the possibility for rare longer distance movements in continuous habitats. Where remnant populations of *D. elastica* are small and highly fragmented, we predict that there will be little pollen movement between these remnant populations (>1 km apart), given the inability of the agricultural matrix to support *Z. gilesi*. Direct investigation of orchid pollen movement will be required to validate these predictions and understand population connectivity via pollen flow in this species. Further, the long-distance movements of these wasps may provide the opportunity for long-distance pollen movement of the food plants. It would be interesting to combine observation of food-foraging and mate-searching to elucidate their relative roles in pollen movement for both orchids and food plants.

Knowledge of the behavioural ecology of these wasps is needed to understand how movements interact with habitat and nectar resource use at the landscape scale. Capture-mark-recapture studies of pollinators are usually focused on the local-scale and by themselves do not necessarily provide information of landscape-scale movements. Resolving patterns of landscape use could be achieved through the use of radio-telemetry, where recent advances leading to miniaturisation of transmitters has allowed long distance movements of individual pollinators to be tracked [47]-[49]. However, commercially available radio-tags are not yet small enough for application on many pollinator species.

The potential for habitat requirements to vary across multiple spatial scales will have implications for population dynamics of these wasps and ultimately orchid pollen movement and fruit set. For example, at the local scale, populations may be dependent on the presence of nectar plants, whereas at the landscape scale, occupancy may be affected by the proportion of remnant vegetation. Further insight into landscape use and foraging movements would require the study of copulating pairs and individuals at food sources. If large-bodied thynnines move relatively long distances to forage on nectar, this may offer the opportunity to connect fragmented populations of both plants and pollinators through the provision of habitat corridors containing nectar-producing plants [28], [50], [51].
